# The BioScope corpus: biomedical texts annotated for uncertainty, negation and their scopes

**DOI:** 10.1186/1471-2105-9-S11-S9

**Published:** 2008-11-19

**Authors:** Veronika Vincze, György Szarvas, Richárd Farkas, György Móra, János Csirik

**Affiliations:** 1 University of Szeged, Department of Informatics, Human Language Technology Group, Árpád tér 2., Szeged, Hungary; 2 Research Group on Artificial Intelligence of the Hungarian Academy of Sciences and University of Szeged, Aradi Vértanúk tere 1., Szeged, Hungary

## Abstract

**Background:**

Detecting uncertain and negative assertions is essential in most BioMedical Text Mining tasks where, in general, the aim is to derive factual knowledge from textual data. This article reports on a corpus annotation project that has produced a freely available resource for research on handling negation and uncertainty in biomedical texts (we call this corpus the BioScope corpus).

**Results:**

The corpus consists of three parts, namely medical free texts, biological full papers and biological scientific abstracts. The dataset contains annotations at the token level for negative and speculative keywords and at the sentence level for their linguistic scope. The annotation process was carried out by two independent linguist annotators and a chief linguist – also responsible for setting up the annotation guidelines – who resolved cases where the annotators disagreed. The resulting corpus consists of more than 20.000 sentences that were considered for annotation and over 10% of them actually contain one (or more) linguistic annotation suggesting negation or uncertainty.

**Conclusion:**

Statistics are reported on corpus size, ambiguity levels and the consistency of annotations. The corpus is accessible for academic purposes and is free of charge. Apart from the intended goal of serving as a common resource for the training, testing and comparing of biomedical Natural Language Processing systems, the corpus is also a good resource for the linguistic analysis of scientific and clinical texts.

## Background

Detecting uncertain and negative assertions is essential in most Text Mining tasks where, in general, the aim is to derive factual knowledge from textual data. This is especially so for many tasks in the biomedical (medical and biological) domain, where these language forms are used extensively in textual documents and are intended to express impressions, hypothesised explanations of experimental results or negative findings. Take, for example, the clinical coding of medical reports, where the coding of a negative or uncertain disease diagnosis may result in an over-coding financial penalty. Another example from the biological domain is interaction extraction, where the aim is to mine text evidence for biological entities with certain relations between them. Here, while an uncertain relation or the non-existence of a relation might be of some interest for an end-user as well, such information must not be confused with real textual evidence (reliable information). A general conclusion is that for text mining, extracted information that is within the scope of some negative/speculative (hedge or soft negation) keyword should either be discarded or presented separately from factual information.

Even though many successful text processing systems [1-3] handle the above-mentioned phenomena, most of them exploit hand-crafted rule-based negation/uncertainty detection modules. To the best of our knowledge, there are no publicly available standard corpora of reasonable size that are usable for evaluating the automatic detection and scope resolution of these language phenomena. The availability of such a resource would undoubtedly facilitate the development of corpus-based statistical systems for negation/hedge detection and resolution.

Our study seeks to fill this gap by presenting the BioScope corpus, which consists of medical and biological texts annotated for negation, speculation and their linguistic scope. We created the corpus to permit a comparison between and to facilitate the development of systems for negation/hedge detection and scope resolution. The corpus described in this paper has been made publicly available for research purposes and it is freely downloadable from .

### Related work

Chapman et al. [2] created a simple regular expression algorithm called NegEx that can detect phrases indicating negation and identify medical terms falling within the negative scope. With this process, a large part of negatives can be identified in discharge summaries.

Mutalik et al. [4] earlier developed Negfinder in order to recognise negated patterns in medical texts. Their lexer uses regular expressions to identify words indicating negation and then it passes them as special tokens to the parser, which makes use of the single-token look-ahead strategy. Thus, without appealing to the syntactic structure of the sentence, Negfinder can reliably identify negated concepts in medical narrative when they are located near the negation markers.

Huang and Lowe [5] implemented a hybrid approach to automated negation detection. They combined regular expression matching with grammatical parsing: negations are classified on the basis of syntactic categories and they are located in parse trees. Their hybrid approach is able to identify negated concepts in radiology reports even when they are located at some distance from the negative term.

The Medical Language Extraction and Encoding (MedLEE) system was developed as a general natural language processor in order to encode clinical documents in a structured form [1]. Negated concepts and certainty modifiers are also encoded within the system, thus it enables them to make a distinction between negated/uncertain concepts and factual information which is crucial in information retrieval.

Elkin et al. [3] use a list of negation words and a list of negation scope-ending words in order to identify negated statements and their scope.

Although a fair amount of literature on uncertainty (or hedging) in scientific texts has been produced since the 1990s (e.g. [6]), speculative language from a Natural Language Processing perspective has only been studied in the past few years. Previous studies [7] showed that the detection of hedging can be solved effectively by looking for specific keywords which imply speculative content.

Another possibility is to treat the problem as a classification task and train a statistical model to discriminate speculative and non-speculative assertions. This approach requires the availability of labeled instances to train the models on. Medlock and Briscoe [8] proposed a weakly supervised setting for hedge classification in scientific texts where the aim is to minimise human supervision needed to obtain an adequate amount of training data. Their system focuses on locating hedge cues in text and thus they do not determine the scopes (in other words in a text they define the scope to be a whole sentence).

### Related resources

Even though the problems of negation (mainly in the medical domain) and hedging (mainly in the scientific domain) have received much interest in the past few years, open access annotated resources for training, testing and evaluation studies are rare and relatively small in size. Our corpus is the first one with an annotation of both negative/speculative keywords and their scope. The authors are only aware of the following related corpora:

1. The Hedge classification corpus [8], which has been annotated for hedge cues (at the sentence level) and consists of five full biological research papers (1537 sentences). No scope annotation is given in the original corpus. We included this publicly available corpus in ours, enriching the data with annotation for negation cues and linguistic scope for both hedging and negation

2. The Genia Event corpus [9], which annotates biological events with negation and three levels of uncertainty (1000 abstracts).

3. The BioInfer corpus [10], where biological relations are annotated for negation (1100 sentences in size).

In the two latter corpora biological terms (relations and events) have been annotated for both negation and hedging, but linguistic cues (i.e. which keyword modifies the semantics of the statement) have not been annotated. We annotated both keywords and their linguistic scope, which is very useful for machine learning or rule-based negation and hedge detection systems.

## Methods

This section describes the basic principles on the annotation of speculative and negative scopes in biomedical texts: basic definitions and general guidelines are illustrated with lots of examples and some special cases and exceptions are also presented. The annotation process of the corpus is also discussed in detail. The document including the annotation guidelines is available from the corpus homepage.

### Basic issues

In a text, sentences with some instance of speculative or negative language only are considered for annotation. The annotation is based on linguistic principles, i.e. parts of sentences which do not contain any biomedical term are also annotated if they assert the non-existence/uncertainty of something.

As for speculative annotation, sentences that state the possible existence of a thing, i.e. neither its existence nor its non-existence is unequivocally stated are considered speculative sentences.  If a sentence is a statement, that is, it does not include any speculative element that suggests uncertainty, it is disregarded. Questions inherently suggest uncertainty – which is why they are asked -, but they will be neglected and not annotated unless they contain speculative language.

Sentences containing any kind of negation are examined for negative annotation. Negation is understood as the implication of the non-existence of something. However, the presence of a word with negative content does not imply that the sentence should be annotated as negative, since there are sentences that include grammatically negative words but have a speculative meaning or are actually regular assertions (see the examples below).

In the corpus, instances of speculative and negative language – that is, keywords and their scope – are annotated. Speculative elements are marked by angled brackets: <*or*>, <*suggests*> etc., while negative keywords are marked by square brackets: [*no*], [*without*] etc. The scope of both negative and speculative keywords is denoted by parentheses. Also, the speculative or negative cue is always included within its scope:

This result (<suggests> that the valency of Bi in the material is smaller than + 3).

Stable appearance the right kidney ([without] hydronephrosis).

In the following, the general guidelines for speculative and negative annotation are presented.

### General guidelines

During the annotation process, we followed a *min-max *strategy for the marking of keywords (*min*) and their scope (*max*). When marking the keywords, a minimalist strategy was followed: the minimal unit that expresses hedging or negation is marked as a keyword. However, there are some cases when a hedge or negation can be expressed via a phrase rather than a single word. Complex keywords are phrases that express uncertainty or negation together, but they cannot do this on their own (the meaning or the semantics of its subcomponents are significantly different from the semantics of the whole phrase). An instance of a complex keyword can be seen in the following sentence:

Mild bladder wall thickening (<raises the question of> cystitis).

On the other hand, a sequence of words cannot be marked as a complex keyword if it is only one of those words that express speculative or negative content (even without the other word). Thus prepositions, determiners, adverbs and so on are not annotated as parts of the complex keyword if the keyword can have a speculative or negative content on its own:

The picture most (<likely> reflects airways disease).

Complex keywords are not to be confused with the sequence of two or more keywords because they can express hedge or negation on their own, that is, even without the other keyword. In this case, each keyword is annotated separately, as is shown in the following example:

Slightly increased perihilar lung markings (<may> (<indicate> early reactive airways disease)).

### Scope marking

When marking the scopes of negative and speculative keywords, we extended the scope to the largest syntactic unit possible (in contrast to other corpora like the one described in [4]). Thus, annotated scopes always have the maximal length – as opposed to the strategy for annotating keywords, where we marked the minimal unit possible. Our decision was supported by two facts. First, since scopes must contain their keywords, it seemed better to include every element in between the keyword and the target word in order to avoid "empty" scopes, that is, scopes without a keyword. In the next example, *however *is not affected by the hedge cue but it should be included within the scope, otherwise the keyword and its target phrase would be separated:

(Atelectasis in the right mid zone is, however, <possible>).

Second, the status of modifiers is occasionally vague: it is sometimes not clear whether the modifier of the target word belongs to its scope as well. The following sentence can describe two different situations:

There is [no] primary impairment of glucocorticoid metabolism in the asthmatics.

First, the glucocorticoid metabolism is impaired in the asthmatics but not primarily, that is, the scope of *no *extends to *primary* only: *([no] primary)*. Second, the scope of *no *extends to *impairment *(and its modifiers and complements as well), thus there is no impairment of the glucocorticoid metabolism at all: *([no] primary impairment of glucocorticoid metabolism in the asthmatics)*. Another example is shown here:

Mild viral <or> reactive airways disease is detected.

The syntactic structure of the above sentence is ambiguous. First, the airways disease is surely mild, but it is not known whether it is viral or reactive: *(viral <or> reactive)*; or second, the airways disease is either mild and viral or reactive and not mild *(mild viral <or> reactive)*. Most of the sentences with similar problems cannot be disambiguated on the basis of contextual information, hence the proper treatment of such sentences remains problematic. However, we chose to mark the widest scope available: in other words, we preferred to include every possible element within the scope rather than exclude elements that should probably be included.  Thus, in the previous two examples, the wider scopes were finally marked.

The scope of a keyword can be determined on the basis of syntax. The scope of verbs, auxiliaries, adjectives and adverbs usually extends to the right of the keyword. In the case of verbal elements, i.e. verbs and auxiliaries, it ends at the end of the clause (if the verbal element is within a relative clause or a coordinated clause) or the sentence, hence all complements and adjuncts are included, in accordance with the principle of maximal scope size. Take the following examples:

The presence of urothelial thickening and mild dilatation of the left ureter (<suggest> that the patient may have continued vesicoureteral reflux).

These findings that (<may> be from an acute pneumonia) include minimal bronchiectasis as well.

These findings (<might> be chronic) and (<may> represent reactive airways disease).

The scope of attributive adjectives generally extends to the following noun phrase, whereas the scope of predicative adjectives includes the whole sentence. For example, in the following two statements:

This is a 3 month old patient who had (<possible> pyelonephritis) with elevated fever.

(Atelectasis in the right mid zone is, however, <possible>).

Sentential adverbs have a scope over the entire sentence, while the scope of other adverbs usually ends at the end of the clause or sentence. For instance,

(The chimaeric oncoprotein <probably> affects cell survival rather than cell growth).

Right upper lobe volume loss and (<probably> pneumonia).

The scope of conjunctions extends to all members of the coordination. That is, it usually extends to the both left and right:

Symptoms may include (fever, cough <or> itches).

Complex keywords such as *either ... or *have one scope:

Mild perihilar bronchial wall thickening may represent (<either> viral infection <or> reactive airways disease).

Prepositions have a scope over the following (noun) phrase:

Mildly hyperinflated lungs ([without] focal opacity).

When the subject of the sentence contains the negative determiners *no *or *neither*, its scope extends to the entire sentence:

Surprisingly, however, ([neither] of these proteins bound in vitro to EBS1 or EBS2).

The main exception that changes the original scope of the keyword is the passive voice. The subject of the passive sentence was originally the object of the verb, that is, it should be within its scope. This is why the subject must also be marked within the scope of the verb or auxiliary. For instance,

(A small amount of adenopathy <cannot be> completely <excluded>).

Another example of scope change is the case of raising verbs (*seem, appear, be expected, be likely *etc.). These can have two different syntactic patterns, as the following examples suggest:

It seems that the treatment is successful.

The treatment seems to be successful.

In the first case, the scope of *seems *starts right with the verb. If this was the case in the second pattern, *the treatment *would not be included in the scope, but it should be like that shown in the first pattern. Hence in the second sentence, the scope must be extended to the subject as well:

It (<seems> that the treatment is successful).

(The treatment <seems> to be successful).

Sometimes a negative keyword is present in the text apparently without a scope: *negative *obviously expresses negation, but the negated fact – what medical problem the radiograph is negative for – is not part of the sentence. In such cases, the keyword is marked and the scope contains the keyword only:

([Negative]) chest radiograph.

In the case of elliptic sentences, the same strategy is followed: the keyword is marked and its scope includes only the keyword since the verbal phrase, that is, the scope of *not*, is not repeated in the sentence.

This decrease was seen in patients who responded to the therapy as well as in those who did ([not]).

Generally, punctuation marks or conjunctions function as scope boundary markers in the corpus, in contrast to the corpus described in [4] where certain lexical items are treated as negation-termination tokens. Since in our corpus the scope of negation or speculation is mostly extended to the entire clause in the case of verbal elements, it is clear that markers of a sentence or clause boundary determine the end of their scope.

### Special cases

It seems unequivocal that whenever there is a speculative or negative cue in the sentence, the sentence expresses hedge or negation. However, we have come across several cases where the presence of a speculative/negative keyword does not imply a hedge/negation. That is, some of the cue candidates do not denote speculation or negation in all their occurrences. In other words, they are ambiguous.

For instance, the following sentence is a statement and it is the emergence of the wandering homozygous larvae form that is stated, and it is not an instance of hedging (although it contains the cue candidate *appear*):

*Development during the third larval instar is significantly delayed, and wandering homozygous larvae usually ****appear *** *2 d after their heterozygous siblings, which start wandering at about 5 d of development.*

As for negative cues, sentences including a negative keyword are not necessarily to be annotated for negation. They can, however, have a speculative content as well. The following sentence contains *cannot*, which is a negative keyword on its own, but not in this case:

(A small amount of adenopathy <cannot be> completely <excluded>).

Some other sentences containing a negative keyword are not to be annotated either for speculation or for negation. In the following example, the negative keyword is accompanied by an adverb and their meaning is neither speculative nor negative. The sequence of the negative keyword and the adverb can be easily substituted by another adverb or adjective having the same (or a similar) meaning, which is by no means negative – as shown in the example. In this way, the sentence below can be viewed as a positive assertion (not a statement of the non-existence of something).

Thus, signaling in NK3.3 cells is not always (=sometimes) identical with that in primary NK cells.

Finally, the problem of intersecting scopes is illustrated by the following example:

Repression did [not] <seem> to involve another factor whose activity is affected by the NSAIDs.

The sentence includes a negative keyword (*not*) and a speculative keyword (*seem*) as well. Following the general guidelines, their scope would be annotated as follows:

(Repression did ([not] <seem> to involve another factor whose activity is affected by the NSAIDs)).

Thus, the scope of *not *starts with the keyword and ends at the end of the sentence, while the scope of *seem *includes the whole sentence. However, this solution seems to pose some further problems. First, the scopes overlap: the negative scope is a subset of the speculative scope. Second, the speculative scope appears to be "empty", that is, it does not include a keyword, and the negative scope contains a negative cue and a speculative one as well. Thus, in these cases, we applied the strategy of scope extension: the negative scope was extended to the whole sentence in order to avoid intersecting scopes, yielding:

((Repression did [not] <seem> to involve another factor whose activity is affected by the NSAIDs)).

As can be seen from the above examples, hedging or negation is determined not just by the presence of an apparent cue: it is rather an issue of the keyword, the context and the syntactic structure of the sentence taken together. On the other hand, scopes can also be extended if it is required by the presence of other keywords.

### The annotation process

Our BioScope corpus was annotated by two independent linguists following the guidelines written by our linguist expert before the annotation of the corpus was initiated. These guidelines were modified several times during the annotation stage as annotators were confronted with new problematic issues. The annotators were not allowed to communicate with each other as far as the annotation process was concerned, but they could turn to the linguist expert when needed and regular meetings were also held between the annotators and the linguist expert in order to discuss recurring and/or frequent problematic issues. When the two annotations for one subcorpus were finalised, differences between the two were resolved by the linguist expert, yielding the gold standard labeling of the subcorpus.

## Discussion

In this section we elaborate on the overall characteristics of the corpus we developed, including a brief description of the texts that constitute the BioScope corpus and some general statistics concerning the size of each part, distribution of negation/hedge cues and ambiguity levels, then we present statistics on the overall results of the annotation work.

### Corpus texts

The corpus consists of texts taken from 4 different sources and 3 different types in order to ensure that it captures the heterogeneity of language use in the biomedical domain. We decided to add clinical free-texts (radiology reports), biological full papers and biological paper abstracts (texts from Genia).

Table 1 summarises the chief characteristics of the three subcorpora. The 3rd and 5th rows of the table show the ratio of sentences which contain negated or uncertain statements. The 4rd and 6th rows show the number of negation and hedge cue occurrences in the given corpus.

**Table 1:** Statistics of the three subcorpora

	Clinical	Full Paper	Abstract
#Documents	1954	9	1273
#Sentences	6383	2670	11871
Negation sentences	13.55%	12.70%	13.45%
#Negation cues	877	389	1848
Hedge sentences	13.39%	19.44%	17.70%
#Hedge cues	1189	714	2769

A major part of the corpus consists of clinical free-texts. We chose to add medical texts to the corpus in order to facilitate research on negation/hedge detection in the clinical domain. The radiology report corpus that was used for the clinical coding challenge [11] organised by the Computational Medicine Center in Cincinatti, Ohio in 2007 was annotated for negations and uncertainty along with the scopes of each phenomenon. This part contains 1954 documents, each having a *clinical history *and an *impression *part, the latter being denser in negated and speculative parts.

Another part of the corpus consists of full scientific articles. 5 articles from FlyBase (the same data were used by Medlock and Briscoe [8] for evaluating sentence-level hedge classifiers) and 4 articles from the open access BMC Bioinformatics website were downloaded and annotated for negations, uncertainty and their scopes. Full papers are particularly useful for evaluating negation/hedge classifiers as different parts of an article display different properties in the use of speculative or negated phrases. Take, for instance, the *Conclusions *section of scientific papers that tends to contain significantly more uncertain or negative findings than the description of *Experimental settings and methods*.

Scientific abstracts are the main targets for various Text Mining applications like protein-protein interaction mining due to their public accessibility (e.g. through PubMed). We therefore decided to include quite a lot of texts from the abstracts of scientific papers. This is why we included the abstracts of the Genia corpus [12]. This decision was straightforward for two reasons. First, the Genia corpus contains syntax tree annotation, which allows a comparison between scope annotation and syntactic structure. Being syntactic in nature, scopes should align with the bracket structure of syntax trees, while scope resolution algorithms that exploit treebank data can be used as a theoretical upper bound for the evaluation of parsers for resolving negative/hedge scopes. The other reason was that scope annotation can mutually benefit from the rich annotations of the Genia corpus, such as term annotation (evaluation) and event annotation (comparison with the biologist uncertainty labeling of events).

The corpus consists of more than 20.000 annotated sentences altogether. We consider this size to be sufficiently large to serve as a standard evaluation corpus for negation/hedge detection in the biomedical domain.

### Agreement analysis

We measured the consistency level of the annotation using inter-annotator agreement analysis. The inter-annotator agreement rate is defined as the F_β = 1 _measure of one annotation, treating the second one as the gold standard. We calculated agreement rates for all three subcorpora between the two independent annotators and between each annotator and the gold standard labeling. The gold standard labeling was prepared by the creator of the annotation guide, who resolved all cases where the two annotators disagreed on a keyword or its scope annotation. Our results are shown in Table 2. The agreement rates between each annotator and the gold standard labeling tell us that there was a high level of agreement between one of the annotators and the linguist expert whereas the agreement rate between the other annotator and the linguist expert was considerably lower. This may be attributed to the fact that the first annotator had more experience in analyzing scientific texts, thus, for her to follow the annotation principles was not such a demanding task than for the other annotator, who previously had little experience in text analysis.

**Table 2:** Agreement rates for the three subcorpora. The chief annotator resolved just the cases where the first two annotators disagreed, cases of agreement were accepted without further checking. The numbers denote agreement between the two student annotators (first one), and the agreements between each student and the chief annotator (second and third numbers).

type		clinical records	abstracts	full articles
NEGATION				
	keyword	90.70/94.56/95.81	91.46/91.71/98.05	79.42/86.77/91.71
	left scope	86.27/86.86/97.95	97.78/97.90/100	83.44/82.42/95.87
	right scope	88.88/91.26/97.39	94.56/95.17/99.42	84.36/88.19/95.09
	full scope	76.29/79.32/95.35	92.46/93.07/99.42	70.86/73.35/91.21

SPECULATION				
	keyword	84.01/89.86/92.37	79.12/83.92/92.05	77.60/81.49/90.81
	left scope	89.36/88.90/97.60	87.52/88.37/97.58	75.49/80.13/92.15
	right scope	91.28/92.64/97.90	87.13/89.92/96.16	82.40/83.28/96.97
	full scope	81.90/82.88/95.54	76.72/80.07/94.04	62.50/66.72/89.67

We measured the agreement rate of annotating negative and hedge keywords, and the agreement rate of annotating the linguistic scope for each phenomenon. We distinguished left-scope, right-scope and full scope agreement that required both left and right scope boundaries to match exactly to be considered as coinciding annotations. A detailed analysis of the consistency levels for the three subcorpora, and the ambiguity levels for the most frequent negative and hedge keyword candidates (that is, the ratio of a keyword being annotated as a negative/speculative cue and the number of occurrences of the same keyword candidate in the corpus) can be found in Tables 2 and 3, in additional file 1 and on the corpus homepage.  A comprehensive list of speculative and negative keywords is also available on the corpus homepage.

**Table 3:** Estimation of consistency in cases of initial agreement. We collected 200-200 randomly chosen examples from each type of corpus text to assess the level of consistency in cases when the two students provided identical annotation for the sentence (identical means here that all cues and scope boundaries were exactly the same) and they were compared to the annotation provided by the chief annotator.  The agreement rates are given here.

NEGATION	
keyword:	98.65%
left scope:	97.27%
right scope:	98.64%
full scope:	95.91%
SPECULATION	
keyword:	99.63%
left scope:	99.25%
right scope:	99.63%
full scope:	98.88%

### BioScope corpus availability

The corpus is available free of charge for research purposes and can be obtained for a modest price for business use. For more details, see the BioScope homepage: .

## Conclusion

In this paper we reported on the construction of a corpus annotated for negations, speculations and their linguistic scopes. The corpus is accessible for academic purposes and is free of charge. Apart from the intended goal of serving as a common resource for the training, testing and comparison of biomedical Natural Language Processing systems, the corpus is also a useful resource for the linguistic analysis of scientific and clinical texts.

The most obvious conclusions here are that the usual language of clinical documents makes it much easier to detect negation and uncertainty cues than in scientific texts because of the very high ratio of the actual cue words (i.e. low ambiguity level), which explains the high accuracy scores reported in the literature. In scientific texts – which are nowadays becoming a popular target for Text Mining (for literature-based knowledge discovery) – the detection and scope resolution of negation and uncertainty is, on the other hand, a problem of great complexity, with the percentage of non-hedge occurrences being as high as 90% for some hedge cue candidates in biological paper abstracts. Take, for example, the keyword *or*, which is labeled as a speculative keyword in only 8.85% of the cases in scientific abstracts, while it was labeled as speculative in 98.08% of the cases in clinical texts. Identifying the scope is also more difficult in scientific texts where the average sentence length is much longer than in clinical data, and the style of the texts is also more literary in the former case.

In our study we found that hedge detection is a more difficult problem than identifying negations because the number of possible cue words is higher and the ratio of real cues is significantly lower in the case of speculation (higher keyword/non-keyword ambiguity). The annotator-agreement table also confirms this opinion: the detection of hedging is more complicated than negation even for humans.

Our corpus statistics also highlight the importance of negation and hedge detection in the biomedical domain. The ratio of negated and hedge sentences in the corpus varies in the subcorpora, but we can say that over 10% of the sentences contains a modifier that radically influences the semantic content of the sentence.

One of the chief construction principles of the BioScope corpus was to facilitate the training/development of automatic negation and hedge detection systems. Such systems have to solve two sub-problems: they have to identify real cue words (note that the probability of any word being a keyword can be different for various domains) and then they have to determine the linguistic scope of actual keywords.

These automatic hedge and negation detection methods can be utilised in a variety of ways in a (biomedical) Text Mining system. They can be used as a preprocessing tool, i.e. each word in a detected scope can be removed from the documents if we seek to extract true assertions. This can significantly reduce the level of noise for processing in the kind of cases where only a document-level labeling is provided (like that for the ICD-9 coding dataset) and just clear textual evidence for certain things should be extracted. On the other hand, similar systems can classify previously extracted statements based on their certainty or uncertainty, which is generally an important issue in the automatic processing of scientific texts.

## Competing interests

The authors declare that they have no competing interests.

## Authors' contributions

Veronika Vincze designed and prepared the annotation guidelines, trained the linguist students who carried out the first annotation phase and then she resolved cases of disagreement. She was responsible for all the linguistic aspects throughout the project.

György Szarvas designed the corpus, collected texts and designed the annotation process and guidelines from a computer science point of view. He was responsible for the Text Mining aspects taken into account in the annotation project (including some general considerations about the annotation like minimal keyword/maximal scope principles), and he designed the evaluation methodology.

Richárd Farkas and János Csirik supervised the work and were involved in the writing of the manuscript. RF was also responsible for the design of the corpus home page and the dissemination of the dataset presented here.

György Móra contributed to the development of the software tools used in the project (evaluation method, data conversion, filtering and validating scripts). He was responsible for all the statistical analysis given in this paper, he provided technical assistance to the annotators and he was also involved in designing the analysis parts of the study.

All the co-authors read and approved the manuscript.

## Supplementary Material

Additional file 1 **Statistics of the top10 keyword candidates for each subcorpus**. We present statistics (#annotated, #not annotated, keyword%, average scope size) for the 10 most common negation and speculative cue candidates for each subcorpus. Click here for file
