# Supplementary material for: The BioScope corpus: biomedical texts annotated for uncertainty, negation and their scopes
Source: BMC Bioinformatics. 2008 Nov 19;9(Suppl 11):S9. doi: 10.1186/1471-2105-9-S11-S9 (PMC2586758; doi:10.1186/1471-2105-9-S11-S9)
Supplement: Additional file 1 — Statistics of the top10 keyword candidates for each subcorpus. We present statistics (#annotated, #not annotated, keyword%, average scope size) for the 10 most common negation and speculative cue candidates for each subcorpus. [file 1471-2105-9-S11-S9-S1.pdf]

Statistics of the top10 keyword candidates for each subcorpus

We present statistics (#annotated, #not annotated, keyword%, average scope size) for the 10 most common negation and speculative cue candidates for each subcorpus.

**CLINICAL**				
NEGATION	#non-annotated	#annotated	neg cue%	avg scope length (tokens)
no	0	672	100%	4
without	0	97	100%	3
not	4	59	94%	5
negative	0	21	100%	0
lack	2	4	67%	6
can not	0	4	100%	4
none	1	2	67%	1
no longer	0	2	100%	2
absence	0	2	100%	3
rule - out	0	2	100%	2
SPECULATION	#non-annotated	#annotated	spec cue%	avg scope length (tokens)
or	5	256	98%	4
evaluate for	0	144	100%	3
rule - out	0	130	100%	2
suggest	0	108	100%	5
may	0	107	100%	7
versus	0	102	100%	4
likely	0	67	100%	5
consistent with	0	67	100%	5
appear	34	24	41%	5
possible	1	43	98%	4

**ARTICLES**				
NEGATION	#non-annotated	#annotated	neg cue%	avg scope length (tokens)
not	37	200	84%	7
no	10	49	83%	7
lack	0	38	100%	5
failed	2	26	93%	8
without	0	26	100%	5
can not	0	24	100%	10
exclude	14	2	13%	1
rather than	0	13	100%	8
absence	5	6	55%	5
none	1	10	91%	14
SPECULATION	#non-annotated	#annotated	spec cue%	avg scope length (tokens)
predict	188	12	6%	15
or	124	18	13%	12
suggest	0	140	100%	13
can	109	20	16%	12
estimate	89	13	13%	13
indicate that	0	80	100%	15

appear	13	65	83%	16
if	69	3	4%	13
may	1	66	99%	11
prediction	63	2	3%	6

ABSTRACTS

NEGATION	#non-annotated	#annotated	neg cue%	avg scope length (tokens)
not	74	1026	93%	7
no	35	205	85%	8
either	152	2	1%	3
failed	26	106	80%	9
lack	0	112	100%	6
absence	38	57	60%	6
without	0	83	100%	5
unable	0	30	100%	10
can not	0	30	100%	9
absent	12	13	52%	7

SPECULATION	#non-annotated	#annotated	spec cue%	avg scope length (tokens)
suggest	4	1226	100%	18
or	937	91	9%	7
may	1	516	100%	12
indicate that	0	509	100%	20
can	333	44	12%	11
appear	41	254	86%	17
could	87	67	44%	12
potential	79	47	37%	9
indicate	66	46	41%	13
whether	1	97	99%	15
